# The Dietary Supplement Creatyl-l-Leucine Does Not Bioaccumulate in Muscle, Brain or Plasma and Is Not a Significant Bioavailable Source of Creatine

**DOI:** 10.3390/nu14030701

**Published:** 2022-02-08

**Authors:** Robin P. da Silva

**Affiliations:** Department of Physiology and Pathophysiology, Max Rady College of Medicine, University of Manitoba, Basic Medical Sciences Building, 745 Bannatyne Avenue, Winnipeg, MB R3E 0J9, Canada; robin.dasilva@umanitoba.ca

**Keywords:** creatine, amino acids, dietary supplement

## Abstract

Creatine is an important energy metabolite that is concentrated in tissues such as the muscles and brain. Creatine is reversibly converted to creatine phosphate through a reaction with ATP or ADP, which is catalyzed by the enzyme creatine kinase. Dietary supplementation with relatively large amounts of creatine monohydrate has been proven as an effective sports supplement that can enhances athletic performance during acute high-energy demand physical activity. Some side effects have been reported with creatine monohydrate supplementation, which have stimulated research into new potential molecules that could be used as supplements to potentially provide bioavailable creatine. Recently, a popular supplement, creatyl-l-leucine, has been proposed as a potential dietary ingredient that may potentially provide bioavailable creatine. This study tests whether creatyl-l-leucine is a bioavailable compound and determines whether it can furnish creatine as a dietary supplement. Rats were deprived of dietary creatine for a period of two weeks and then given one of three treatments: a control AIN-93G creatine-free diet, AIN-93G supplemented with creatine monohydrate or AIN-93G with an equimolar amount of creatyl-l-leucine supplement in the diet for one week. When compared to the control and the creatine monohydrate-supplemented diet, creatyl-l-leucine supplementation resulted in no bioaccumulation of either creatyl-l-leucine or creatine in tissue.

## 1. Introduction

Creatine is a critical energy metabolite that is synthesized from the amino acids arginine, methionine, and glycine via a two-enzyme, interorgan, metabolic pathway [1,2,3]. Creatine serves particularly important function in the muscle and brain and is concentrated in these tissues by a sodium-dependent transporter to levels approximately 100-fold greater than plasma. Via a reaction catalyzed by the enzyme creatine kinase, creatine is in equilibrium with creatine phosphate, which effectively serves to buffer cellular ATP concentrations during periods of acute demand for large amounts of energy such as depolarization or contractile events. Approximately 2 g of creatine is lost from the body per day per 70 kg body weight and this creatine is replaced either endogenous synthesis or by absorption from the diet; creatine is obtained in the diet mainly from meat and dairy products [4].

Creatine monohydrate (CrM) has been used as a sports supplement for decades and there is clear evidence that dietary creatine supplements improve muscle performance during burst-type exercises such as sprinting, track and field events or powerlifting [5]. Numerous animal studies have documented that dietary creatine supplementation can increase total muscle and brain creatine content [1]. Albeit brain creatine content does not increase to the same extent as does muscle in response to dietary CrM supplementation since creatine does not easily cross the blood–brain barrier. Indeed it has been shown, using P-31 NMR, that two weeks of dietary supplementation with creatine increased muscle concentrations of creatine phosphate by approximately 20% in young male subjects [6]. Given the significant literature demonstrating the ergogenic benefit of creatine supplementation it is not surprising that creatine is a major component of the more than 5.4 billion dollar (in 2015) sports supplement industry [7].

**Figure 1:**
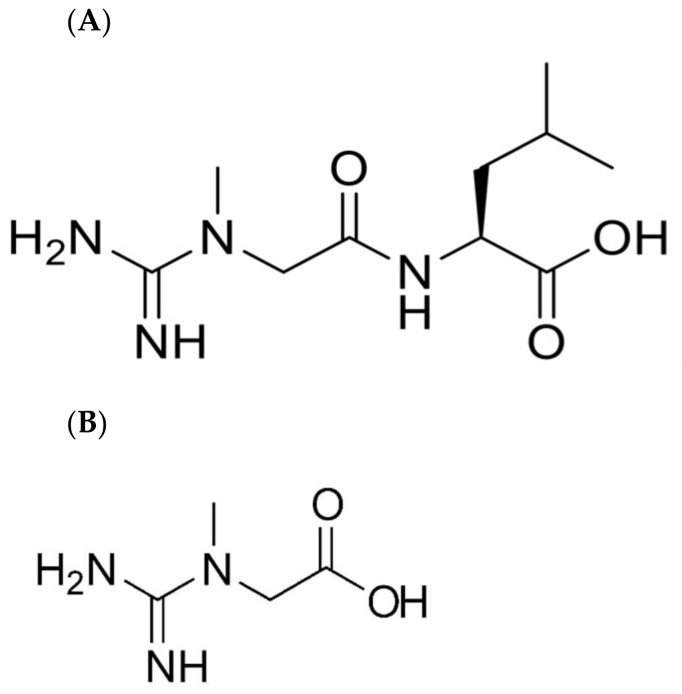
Chemical structures of (**A**) creatyl-l-leucine and (**B**) creatine.

The most common form of dietary creatine supplement is CrM [1,8]. CrM is usually taken as a loading dose of 20 g per day for 5–7 days followed by a maintenance dose of 5 g per day. However, some studies have suggested that the relatively large amount of creatine taken to achieve benefits can cause an osmotic induced gastrointestinal system [1]. This has stimulated some to investigate whether alternate forms of creatine could be better tolerated, stable and bioavailable. Jäger et al. published an extensive analysis of potential sources of creatine and concluded that CrM was the most favorable of the compounds tested [9]. Recently it has been suggested that the molecule creatyl-l-leucine (CLL) (Figure 1) may potentially serve as a bioavailable form of creatine that could be used as a dietary ingredient [10]. In the same study, it was shown that CLL had no toxicity when provided for 90 days at 5 g/kg body weight in rats. However, it was also noted that the biological fate of ingested CLL was unknown. The purpose of the present study is to test the bioavailability of CLL and to determine whether creatine is a digestive or metabolic product of dietary CLL.

## 2. Materials and Methods

All experimental procedures involving animals were conducted by CARE Research LLC in an AAALAC-accredited, USDA-certified, and OLAW-accredited facility. The study design and animal usage were reviewed and approved by the CARE Research Institutional Animal Care and Use Committee (IACUC) for compliance with regulations prior to study initiation (IACUC number 2042). Animal welfare for this study was in compliance with the U.S. Department of Agriculture’s (USDA) Animal Welfare Act (9 CFR Parts 1, 2, and 3), the Guide for the Care and Use of Laboratory Animals, and CARE Research SOPs.

All chemicals were purchased from Sigma-Aldrich, except for HPLC grade Methanol and Formic acid which were purchased from Fisher Scientific. Purified AIN-93G diets [11] that were free of creatine or had supplemented CrM or an equimolar equivalent of CLL were custom ordered by CARE Research, LLC from Harlan Teklad (Envigo). Diets composition can be found in Table 1. The presence of CrM and CLL in the respective experimental diets was verified by aqueous acidic extraction and HPLC analysis as described below.

This study was designed to compare the bioavailability of CLL to that of the common creatine supplement CrM against a creatine-free control diet. To give the most favorable condition for absorption and accretion of creatine or CLL, 24 rats were provided with a control creatine-free AIN-93G diet (CON) for 14 days to deplete body creatine to levels supported by an endogenous synthetic capacity. At day 15, n = 8 rats were randomly chosen and switched to an AIN-93G diet supplemented with 0.4% *w*/*w* CrM (CrMD) and n = 8 different rats were randomly chosen and switched to an AIN-93G diet supplemented with 0.656% *w*/*w* CLL (CLLD). The remaining n = 8 rats were maintained on the creatine-free AIN-93G diet. Experimental diets were fed until tissues were collected on day 22. An equal number of male and female rats were used in each group.

Rats were anesthetized using inhaled isoflurane and blood was collected from the portal vein and abdominal aorta. A portion of the quadricep muscle and the whole brain were removed quickly and snap-frozen in liquid nitrogen. Body weights were recorded every 7 days and food consumption was recorded daily.

Guanidine compounds were analyzed using the method of Buchnerger and Ferdig [12]. Briefly, plasma or tissues were deproteinized using ice-cold perchloric acid and neutralized with potassium carbonate and potassium hydroxide. After deproteinization, the guanidine compounds creatine, guanidinoacetate and CLL were reacted with ninhydrin to produce fluorescent derivatives. These derivatives were separated with an aqueous formic acid and methanol gradient using a Thermo Ultimate UHPLC system equipped with a reverse-phase (C18) HPLC column (Sigma). Pure reference standards were obtained from Sigma-Aldrich, except for CLL which was obtained from Hueston Hennigan LLP.

Validation of CLL measurement was accomplished by derivatizing pure CLL dissolved in water with the method of Buchberger and Ferdig. A distinct peak was observed for CLL that eluted later than creatine. No peak corresponding to CLL was present when a deproteinized water blank or mouse plasma were treated in the same manner. Mouse plasma was spiked with standard CLL and greater than 90% recovery was obtained. A standard curve for CLL was generated to determine the concentration of CLL in samples. The limit of detection for CLL was calculated to be 126 μM and the limit of quantification for CLL was calculated to be 383 μM.

Data were analyzed using a One-way Analysis of Variance (One-way ANOVA) with Tukey’s post hoc test for multiple comparisons. A statistical p value less than 0.05 was used as a cut-off to determine significant statistical differences in means. Linear regression analysis was performed on body weight and food intake data. Data are presented as means +/− one standard deviation from the mean. n = 8 per group.

## 3. Results

**Figure 2:**
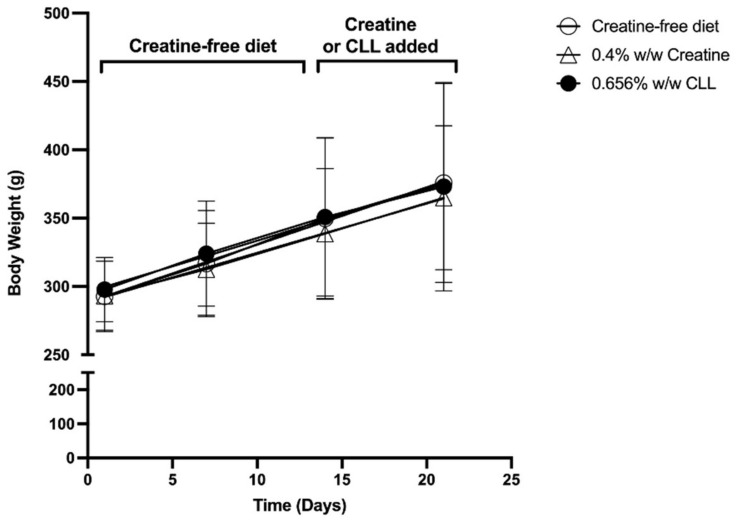
Body weight over the course of the feeding trial. Open circles represent weights for rats fed creatine-free AIN-93G diet for the entire 21 days; open triangles represent weights for rats fed creatine-free diet for 14 days and then switched to AIN-93G diet supplemented with 0.4% (*w*/*w*) creatine monohydrate for the remaining 7 days; black circles represent weights for rats fed creatine-free diet for 14 days and then switched to AIN-93G diet supplemented with 0.656% (*w*/*w*) creatyl-l-leucine (CLL) for the remaining 7 days. Values are presented as the mean ± one standard deviation, n = 8 per group.

**Figure 3:**
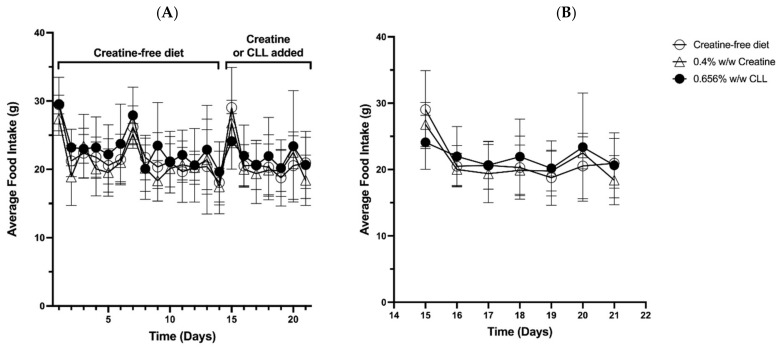
Food intake for all groups. Panel (**A**) Data for all days of experiment and panel (**B**) expanded data for days 15–21. Open circles represent the average daily food intake for rats fed creatine-free AIN-93G diet for the entire 21 days; open triangles represent average daily food intake for rats fed creatine-free diet for 14 days and then switched to AIN-93G diet supplemented with 0.4% (*w*/*w*) creatine monohydrate for days 15–21; black circles represent average daily food intake for rats fed creatine-free diet for 14 days and then switched to AIN-93G diet supplemented with 0.656% (*w*/*w*) creatyl-l-leucine (CLL) for days 15–21. Values are presented as the mean ± one standard deviation, n = 8 per group.

Rats in all groups gained weight and there was no statistical difference in body weight between groups (Figure 2). Food intake was not different between any of the groups (Figure 3) and the average food intake over the course of the experiment was 21.7 ± 3.0, 21.0 ± 2.7 and 22.6 ± 2.4 g per day for the control, CrM and CLL diets, respectively. This amounts to an average daily intake of 0.56 mmoles of creatine by the CrMD rats and 0.61 mmoles of CLL by the CLLD group. For the last two days of the feeding period the rats consumed an average of 0.55 moles of creatine per day in the CrMD group and 0.59 mmoles of CLL per day in the CLLD group. Thus, rats consumed approximately equimolar quantity of creatine and CLL during the feeding period.

**Figure 4:**
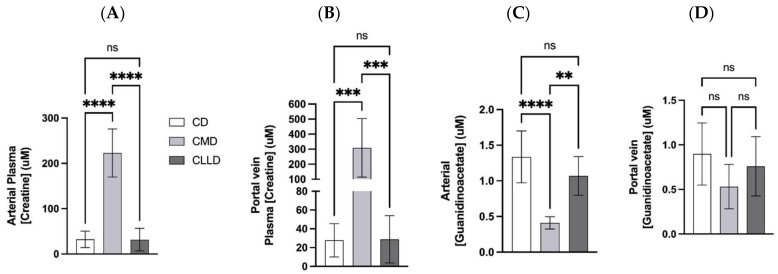
Plasma concentrations of creatine and guanidinoacetate. Panel (**A**) arterial plasma concentration of creatine; panel (**B**) portal venous plasma concentration of creatine; panel (**C**) arterial plasma concentration of guanidinoacetate; panel (**D**) portal venous concentration of guanidinoacetate. The open bar represents rats fed creatine-free diet (CON), light gray bar represents rats fed 0.4% *w*/*w* creatine monohydrate supplemented diet, dark gray bar represents rats fed 0.656% CLL supplemented diet. Values are presented as the mean ± one standard deviation, n = 8 per group. Asterisks indicate significance by *p*-value; *p* < 0.0001 = ****, *p* < 0.001 = ***, *p* < 0.01 = **, ns = not significant.

The creatine concentration in plasma from the abdominal aorta (AA) was approximately 7-fold higher in CrMD-fed rats when compared to either CON or CLLD groups (Figure 4A). Creatine content of portal venous (PV) plasma was 10-fold higher in CMD rats compared to CON and CLLD rats (Figure 4B). The content of creatine in AA and PV plasma from CON and CLLD groups did not differ.

Guanidinoacetate (GAA) is the immediate precursor to creatine that is synthesized from arginine and glycine, primarily in the kidney. GAA can then undergo methylation to form creatine, primarily in the liver [3]. Renal synthesis of GAA is downregulated by dietary creatine supplementation, and we observe that GAA concentration in the AA plasma of CrMD rats was approximately one-third that of CON and CLLD (Figure 4C). GAA in the PV plasma was not significantly different between groups (Figure 4D).

**Figure 5:**
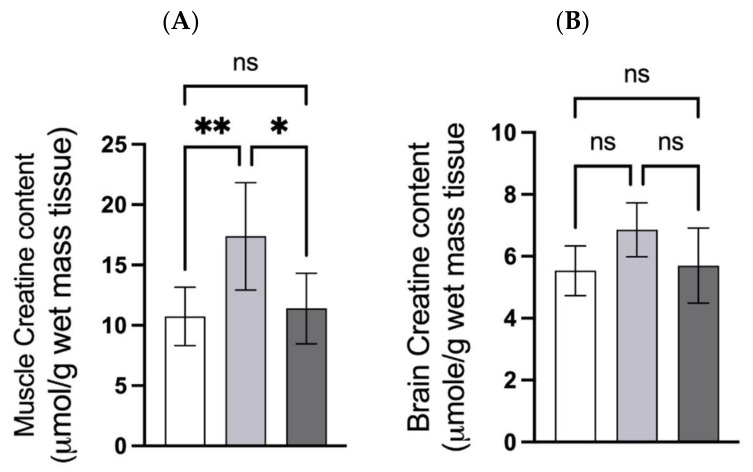
Muscle and brain creatine content. Panel (**A**) Creatine content of quadricep muscle; panel (**B**); creatine content of whole brain. The open bar represents rats fed creatine-free diet (CON), light gray bar represents rats fed 0.4% *w*/*w* creatine monohydrate-supplemented diet, dark gray bar represents rats fed 0.656% CLL supplemented diet. Values are presented as the mean ± one standard deviation, n = 8 per group. Asterisks indicate significance by *p*-value; *p* < 0.01 = **, *p* < 0.05 = *, ns = not significant.

The creatine content of the quadricep muscle from CMD rats was 1.63-fold higher than in CON rats and 1.53-fold higher than in CLLD rats (Figure 5A).

The creatine content of the brain was not significantly different between groups (Figure 5B). However, multiple comparison analysis gave an adjusted P-value of 0.052 (1.26-fold difference in mean) between the CON and CrMD rats, indicating a trend toward higher creatine content in the brain of rats supplemented with CrM.

CLL was not detectable in any of the samples assayed.

## 4. Discussion

CrM remains one of the most widely used dietary supplements that provides significant ergogenic benefit during sport [5]. CrM supplements are also used as a therapy to treat several muscular and neurodegenerative disorders [13,14,15]. CrM is a stable powder that is soluble in water but is somewhat unstable in solution, depending on temperature and pH [1]. Scientific consensus is that CrM as a supplement is safe and the only adverse effect reported was gastrointestinal discomfort [1,16]. Efforts have been made to identify an alternative supplement that has more favorable characteristics and could provide bioavailable creatine. Although several creatine salts have been assessed for favorable properties and bioavailability, CrM was still found to be the most favorable form of creatine supplement [9]. Covalent modification of creatine has also been used to produce new compounds that could potentially be converted to creatine in vivo. The ethyl ester of creatine has been synthesized and tested for bioavailability and muscle performance in humans [17]. However, it was concluded that the ethyl ester of creatine did not increase muscle creatine content or enhance physical performance. CLL has been tested for toxicity and is used as an additive in some sports supplements and beverages under the trademark Super Creatine [18]. The present study is the first controlled study to independently test the bioavailability of CLL and to what extent CLL could be a bioavailable form of creatine.

The intent of this study was to provide the most favorable condition for the absorption and tissue uptake of the test compounds. Provision of a creatine-free diet reduces body creatine stores to a basal level that is sustained by endogenous synthesis of creatine from the amino acids, arginine, glycine, and methionine. Thereafter, relatively high doses of creatine or an equimolar amount of CLL were provided in the diet; a controlled design that compares both compounds equally. It was found that CLL supplementation in the diet did not increase plasma, muscle or brain creatine levels after 7 days of supplementation. In comparison, CrM supplementation significantly increased creatine levels in plasma and muscle while brain creatine content displayed a trend toward an increase. Since the portal vein drains blood from the intestine, it is expected that compounds that are well absorbed from the intestine would be more concentrated in plasma from this vessel. The observation that portal venous plasma creatine concentration from CLLD rats was not different from CON rats indicates that CLL is not converted to creatine in the intestine during digestion. Unpublished data suggested creatyl-L-glutamine was only 27% hydrolyzed to creatine in an in vitro model of digestion [10]. However, we conclude CLL is not a source of creatine during digestion as there was no bioaccumulation of creatine in tissues over the 7 days of CLL supplementation. Moreover, CLL was not detected in the portal venous plasma indicating that CLL is not well absorbed through the intestine. The limit of detection of CLL using the HPLC assay was 126 uM, therefore it cannot be concluded that CLL is not absorbed. However, there was no bioaccumulation of CLL detected in our study indicating that CLL is poorly absorbed even under high levels of dietary supplementation. For comparison, the level of supplement provided in our study would equate to a 70 kg human consuming approximately 17.6 g of creatine or 28.9 g of CLL per day.

Peptide bonds are a similar bond to that found in CLL. Peptide bonds are normally stable under physiological conditions, only hydrolyzed by protease or peptidase enzymes. However, the chemical structure of CLL is not likely to be recognized by the active sites of a protease or peptidase enzymes given the very different structure of creatine when compared to amino acid residues in a peptide. Thus, it is highly unlikely that a peptidase enzyme or other enzyme would bind to CLL to catalyze hydrolysis to creatine and leucine. If an appreciable amount of CLL is either hydrolyzed to creatine and then absorbed, or absorbed and then hydrolyzed in tissues, it would be reasonable to expect to see a significant increase in plasma or tissue creatine concentrations upon dietary supplementation with high doses of CLL, but this was not observed.

In summary, CLL has been proposed as a potential source of creatine when supplemented in the diet and this study tested the bioavailability of CLL in this capacity. The previous literature examining CLL did not test the bioavailability or offer insight into the biological fate of CLL [10,18]. It is now found that rats provided with large doses of CLL in the diet did not yield increases in creatine concentrations in plasma, muscle or brain tissue. CLL was also not detectable in any of the biological samples assayed from rats supplemented with CLL. Thus, it is concluded that CLL did not bioaccumulate, is poorly absorbed by the intestine and is not a bioavailable source of creatine. These data would also suggest that analogous creatine, amino acid-amides, would also have similar properties as dietary supplements.

## Figures and Tables

**Table 1:** Composition of experimental diets.

	CON	CrMD *	CLLD *
Ingredient	g/Kg Diet	g/Kg Diet	g/Kg Diet
Casein	200.0	200.0	200.0
L-Cystine	3.0	3.0	3.0
Corn starch	397.49	393.49	390.93
Maltodextrin	132.0	132.0	132.0
Sucrose	100.0	100.0	100.0
Soybean oil	70.0	70.0	70.0
Cellulose	50.0	50.0	50.0
Mineral Mix (AIN-93G-MX, 94046)	35.0	35.0	35.0
Vitamin Mix (AIN-93-VX, 94047)	10.0	10.0	10.0
Choline bitartrate	2.5	2.5	2.5
Tert-butylhydroquinone	0.014	0.014	0.014
Creatine monohydrate	0	4.0	0
Creatyl-l-leucine	0	0	6.56

* 0.4% Creatine monohydrate (CrMD); 0.656% creatyl-l-leucine (CLLD). American Institute of Nutrition (AIN).

## Data Availability

Data are available upon request.
